# Deep Learning-Aided Modulation Recognition for Non-Orthogonal Signals

**DOI:** 10.3390/s23115234

**Published:** 2023-05-31

**Authors:** Jiaqi Fan, Linna Wu, Jinbo Zhang, Junwei Dong, Zhong Wen, Zehui Zhang

**Affiliations:** 1 School of Cyberspace Science and Technology, Beijing Institute of Technology, Beijing 100081, China; fanjiaqi@bit.edu.cn (J.F.); dongjunwei@bit.edu.cn (J.D.); 2 Aerospace System Engineering Shanghai, Shanghai 201108, China; wulinna1214@sina.com; 3 Science and Technology on Communication Networks Laboratory, The 54th Research Institute of CETC, Shijiazhuang 050081, China; zhjinbo@aliyun.com; 4 Laboratory of Electromagnetic Space Cognition and Intelligent Control, Beijing 100083, China

**Keywords:** automatic modulation recognition, non-orthogonal signal, deep learning, BiLSTM, transfer learning, attention mechanism, spatio-temporal fusion

## Abstract

Automatic Modulation Recognition (AMR) can obtain the modulation mode of the received signal for subsequent processing without the assistance of the transmitter. Although the existing AMR methods have been mature for the orthogonal signals, these methods face challenges when deployed in non-orthogonal transmission systems due to the superimposed signals. In this paper, we aim to develop efficient AMR methods for both downlink and uplink non-orthogonal transmission signals using deep learning-based data-driven classification methodology. Specifically, for downlink non-orthogonal signals, we propose a Bi-directional Long Short-Term Memory (BiLSTM)-based AMR method that exploits long-term data dependence to automatically learn irregular signal constellation shapes. Transfer learning is further incorporated to improve recognition accuracy and robustness under varying transmission conditions. For uplink non-orthogonal signals, the combinatorial number of classification types explodes exponentially with the number of signal layers, which becomes the major obstacle to AMR. We develop a spatio-temporal fusion network based on the attention mechanism to efficiently extract spatio-temporal features, and network details are optimized according to the superposition characteristics of non-orthogonal signals. Experiments show that the proposed deep learning-based methods outperform their conventional counterparts in both downlink and uplink non-orthogonal systems. In a typical uplink scenario with three non-orthogonal signal layers, the recognition accuracy can approach 96.6% in the Gaussian channel, which is 19% higher than the vanilla Convolution Neural Network.

## 1. Introduction

Automatic Modulation Recognition (AMR) analyzes non-cooperative received signals to obtain their modulation types through a series of processes, including signal pre-processing, feature extraction, and classification recognition [1]. AMR has been widely used in various fields, such as cognitive defined radio, military intelligence, communication jammers, surveillance, spectrum management, and communication reconnaissance [2], which can improve spectrum utilization and solve the problem of spectrum shortage. At present, the main goal of the AMR is to quickly and accurately identify the modulation type of the signal for demodulation and analysis, which is an important process for accurately learning and reliably sharing the spectrum to improve the efficiency of spectrum utilization [3].

Conventional modulation recognition is mainly accomplished via sophisticatedly designed signal processing methods [4]. The design of a modulation classifier involves two major aspects: signal pre-processing and modulation classification. Signal pre-processing can combat the unintended channel variations, e.g., noise suppression, matched filtering, and equalization, as well as providing rough parameter estimations, e.g., the Signal-to-Noise Ratio (SNR) and carrier frequency. After pre-processing, classification algorithms can be deployed, which are divided into two categories [5]: likelihood ratio-based algorithms and feature-extraction-based algorithms. The algorithm based on likelihood ratio regards AMR as a multivariate hypothesis testing problem, and uses the likelihood function of the signal as the feature vector, which is then fed into the classifier to form the decision criterion [6]. The algorithm based on feature extraction is similar to the multivariate pattern recognition problem, mapping from the observed signal space to the feature space and then to the decision space [7]. In [8], Jajoo et al. proposed a recognition method based on constellation graph feature clustering for PSK/QAM modulation recognition in slow flat fading channels. In [9], based on the cumulative distribution function curve of the received signal’s normalized instantaneous amplitude, a signal modulation recognition algorithm for a coherent optical receiver is proposed. However, the above classification methods rely on hand-designed criteria or features, which cause poor regularity and adaptability when facing complicated and varying signal models [10].

The recent boom in deep learning (DL) has shed new light on AMR due to its data-driven nature and automatic feature extraction ability [11]. Starting from the raw data end-to-end, features can be automatically extracted using deep neural networks (DNN) with a large number of free parameters, which can avoid the problems of constellation mapping recognition algorithms [12]. A DNN framework is designed in [13] for signature classification based on automatic features, which can complete the signature classification for Internet-of-Things devices. Convolutional Neural Network (CNN) is one of the most popular and successful deep learning architectures. Two layers of CNN are combined in [14] for training on different datasets to achieve high precision discrimination between 16QAM and 64QAM. Tekbıyık et al. propose a novel convolutional neural network (CNN) classifier mode to classify modulation classes in terms of types [15]. The proposed classifier is robust against realistic wireless channel impairments with training datasets for appropriate modeling of real-world conditions. In [16], a improved narrow 2D CNN is applied in large and densely encoded time series for blind temporal learning. Compared with CNN, Recurrent Neural Network (RNN) can process a series of vectors over time and extract PRI better. Considering the sequence characteristics of PRI, RNN combined with an attention mechanism is proposed to identify the modulation type of radar signals [17]. With the representation learning, Zhang et al. proposed an IQ-FOC data representation of the preprocessed signal, where the raw IQ data are combined with the fourth-order cumulant (FOC) of the signal [18]. The IQ-FOC representation enables LSTM models to achieve high classification accuracy. To solve the long-term dependence problem, a Bi-directional Long Short-Term Memory (BiLSTM) layer is cascaded in [19], which is constructed to extract the context information of the signal. In this aspect of combinational neural networks, CNN and LSTM are combined in [20] to extract signal IQ time series features, and the cross-layer link method is used to avoid the loss of effective information. In other works, shallow convolutional networks, Gate Recurrent Unit (GRU), and DNN are combined into CGDNet [21]. Shallow convolutional networks and GRU can extract the features of IQ sequence signals and DNN can complete the classification task after inputting the features.

In the upcoming era of the integration of space, air, and ground networks, the non-orthogonal superposition among radio signals will become a major trend [22,23]. On the one hand, with the intentional introduction of non-orthogonal transmissions, such as non-orthogonal multiple access (NOMA), system throughput and large connectivity can be achieved [22]. On the other hand, with the ever-increasing number of wireless devices and the complicated wireless electromagnetic environment, the superposition among radio signals also becomes inevitable [24]. Non-orthogonal wireless systems are further divided into downlink and uplink scenarios, respectively. The main characteristics of the downlink non-orthogonal systems are single-point transmission and multi-point reception, with a total power limitation. In non-orthogonal uplink transmissions, multiple users simultaneously transmit signals to a receiver using superposition coding. The combinatorial number of signals explodes exponentially under such circumstances. To effectively exploit the spectrum resources in such complicated electromagnetic environments, efficient AMR methods for non-orthogonal systems are required.

Nevertheless, few studies have focused on the aforementioned issue. Dong et al. use a Fully Connected Deep neural Network (FC-DNN) and LSTM to process the multiple received signal packets of NOMA using feedforward and recurrent structures [25]. In [26], a novel machine learning based algorithm is proposed to achieve an automatic modulation classifier and improve the performance of beyond fifth generation (B5G) wireless communication system, which is assisted by Orthogonal Frequency Division Multiplexing (OFDM) and Non-Orthogonal Multiple Access (NOMA) techniques. In order to mine the effective features with a limited number of labels, Pan et al. propose a multi-instance, multi-label, weakly supervised learning method based on GAN [27], taking aliased signals as an example. Nevertheless, there is still a need for efficient AMR methods for uplink and downlink non-orthogonal wireless systems. The major design challenges are laid out as follows:

- For the downlink non-orthogonal scenario, the superposition of signals can cause irregular signal shapes. The conventional hand-crafted features become inefficient. This poses a challenge to both the feature design and signal classification stages since the learning ability of the conventional classifier is limited.
- For uplink non-orthogonal scenarios, the challenge is more significant. With the increase in the transmit signal layers, the combinatorial number of classification types explodes exponentially. Traditional classification methods thus suffer from high computational complexity and low recognition rate.

To tackle the above challenges, this paper aims to exploit the advances in automatic feature extraction and efficient classification ability of DL models to improve the accuracy of AMR for non-orthogonal wireless systems. The contributions of this paper are detailed as follows:

- In the downlink scenario, we propose a modulation recognition method based on BiLSTM to extract the sequential information of the superimposed signals. For the scenario where the number of training samples is insufficient, transfer learning is used to improve the network modulation recognition ability in small sample scenarios.
- In the uplink scenario, we consider Spatio-Temporal Fusion Network based on Attention Mechanism (STFAN), which can deal with the explosive increase of the combinatorial number of classification types. The spatial feature extraction module uses an Inception block to efficiently reduce the computational complexity, and the temporal feature extraction module uses BiLSTM to mine the effective signal features in the time domain.
- Experiments show that the proposed AMR methods for non-orthogonal signals outperform the conventional methods as well as vanilla DL methods, such as CNN and LSTM, under various channel conditions. Significant advantages of the proposed methods with respect to both recognition accuracy and wireless channel robustness are observed, especially in a high SNR region.

The rest of this paper is organized as follows. Section 2 illustrates the model for downlink and uplink non-orthogonal systems. Section 3 and Section 4 analyze the proposed AMR methods for downlink and uplink non-orthogonal signals, respectively. In Section 5, experiments are provided to validate the efficiency of the proposed methods. Finally, Section 6 concludes the paper.

## 2. Signals Model for Non-Orthogonal Transmission Systems

This section elaborates on the downlink and uplink non-orthogonal transmission systems, respectively.

### 2.1. Downlink Non-Orthogonal System Model

In the downlink non-orthogonal system, the base station uses NOMA mapping to superimpose the signals of multiple users on the same time-frequency resource and transmit them. The key design elements of NOMA can be roughly classified into three categories, namely bit-level, symbol-level, and wave-level designs [28]. Based on NOMA adopted by the base station, the user side can select the corresponding receiving algorithm to demodulate and decode the received signal. In the downlink scenario, different user signals can experience the same channel.

**Figure 1:**
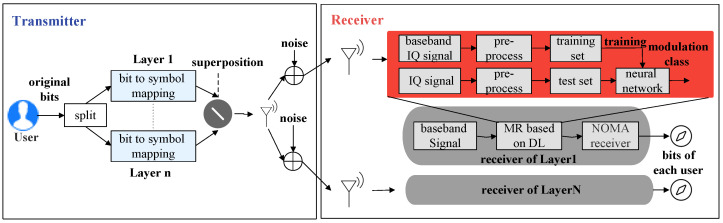
Downlink non-orthogonal system structure.

The overall architecture of the non-orthogonal signal modulation identification system for downlink scenarios is illustrated in Figure 1. The original information bits are transmitted as NOMA symbols after bit-to-symbol mapping on the base station side. The bit-to-symbol mapping includes two parts: constellation mapping and NOMA modulation. This work is mainly for the identification of non-orthogonal schemes; the constellation mapping method is QPSK. On the user side, the baseband symbol sequence is obtained by analog-to-digital conversion and match-filtering. For one of the users, the signal is received through the antenna, and the baseband signal is obtained after downconversion. Then modulation recognition methods based on DL are used to identify the non-orthogonal scheme adopted by the base station. Finally, the corresponding receiver is used to demodulate and decode the output information bits.

Denote h(n) as the channel parameters, then the channel *h*(*n*) between the transmitter and receiver can be simulated using additive white Gaussian noise (AWGN). Linearly increase wideband noise, with constant spectral density and Gaussian amplitude. Denote v(n) as the channel noise, then the baseband symbol sequence obtained after pre-processing can be expressed as

(1) $$y\left(n\right)=h\left(n\right)\left(\sum_{j=1}^{J}\sqrt{P_{j}}x_{j}\left(n\right)\right)+v\left(n\right),$$

where $x_{j}\left(n\right)$ is the sequence of symbols sent by the *j*-th stream, and $P_{j}$ is the transmit power of the *j*-th stream, satisfies $\sum_{j=1}^{J}P_{j}=1$.

In the downlink non-orthogonal system, this work will classify the five non-orthogonal schemes, Sparse Code Division Multiple Access (SCMA), Multi-User Shared Access (MUSA), Polarization Division Multiple Access (PDMA), Power Domain Non-orthogonal Multiple Access (PD-NOMA), and Welch-bound Spreading Multiple Access (WSMA), for modulation identification. The above five multi-user access modes define multi-user bit-to-symbol mapping which can be regarded as modulation. Moreover, each user has its corresponding codeword, and the codeword is in one-to-one correspondence with the subcarrier. Therefore, a strong relationship is contained between each codeword of different modulation recognition methods in the downlink system. The modulation recognition method needs to mine the sequence information. After determining the modulation mode, the weak user demodulates its own symbol by using a Maximum Likelihood detector to treat other user signals as interference.

### 2.2. Uplink Non-Orthogonal System Model

In uplink non-orthogonal scenarios, multiple users transmit signals to the base station separately, which will receive and process the superimposed signals. At the receiver, the baseband signals are received and obtained after downconversion. Then the users are sorted by SINR and demodulated through the serial interference cancellation algorithm.

**Figure 2:**
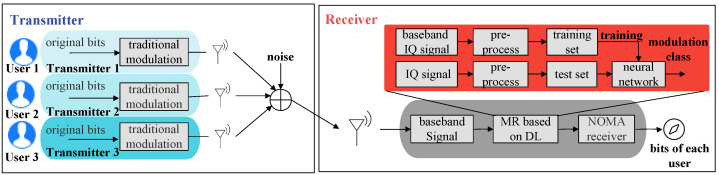
Uplink non-orthogonal system structure.

The structure of an uplink non-orthogonal system is illustrated in Figure 2. The modulation recognition module can obtain the signal category and corresponding power level of each user by identifying the baseband symbol sequence obtained through pre-processing. After that, the bit stream of different users can be recovered through the multiuser non-orthogonal signal receiver using the serial interference removal module, which can demodulate the user signal with the largest power according to the power size. The demodulated data will be deleted from the original data, and the above steps will continue until the data of the user with the minimum power are also demodulated successfully.

Assume that users with different distances from the base station send data to the base station at the same time, the received signal at the base station can be denoted by

(2) $$y\left(n\right)=\sum_{i=1}^{I}h\left(n\right)\sqrt{P_{i}}x_{i}\left(n\right)+v\left(n\right),$$

where h(n) is the channel parameters of the signals sent by the *i*-th user, $x_{i}\left(n\right)$ is the symbol sequences sent by *i*-th user, y(n) is the signal received at the base station, and $\sqrt{P_{i}}$ is the transmit power of *i*-th user. v(n) is the channel white Gaussian noise, which satisfies the expression $n=\sum_{i=1}^{I}v_{i}\left(n\right)$.

In the traditional uplink transmission methods, the signaling is used to identify the modulation mode of each user. After that, SIC will be performed at the receiver, and the users are sorted by SINR and demodulated through the serial interference cancellation algorithm. Due to the explosive growth of the number of modulation modes and limited spectrum resources, modulation recognition methods need to consider reducing signaling overhead.

## 3. Proposed Deep Transfer Learning Incorporated BiLSTM for Downlink AMR

In the downlink non-orthogonal system scenario, the user side needs to know the non-orthogonal scheme used by the base station to determine the receiver. Therefore, this section will focus on the identification of non-orthogonal multiple access schemes in non-orthogonal modulation, and a non-orthogonal signal recognition model based on BiLSTM is proposed. In Section 3.1, we consider the network design process and design the network parameters. The modulation recognition method will be introduced in Section 3.2, based on deep transfer learning for the insufficient number of samples.

### 3.1. Neural Network Architecture with BiLSTM

In the downlink system, the NOMA signal is complex and variable. To mine the information on the sequence of the NOMA signal, a time series model is needed. RNN has the problem of gradient disappearance, which results in the loss of information from previous signal frames when dealing with long sequence data.LSTM can solve the vanishing gradient problem of RNN but cannot encode signal frames that have no direction from backward to forward. BiLSTM is a combination of forward LSTM and backward LSTM, which can extract the information before and after the complex NOMA signal sequences effectively.

**Figure 3:**
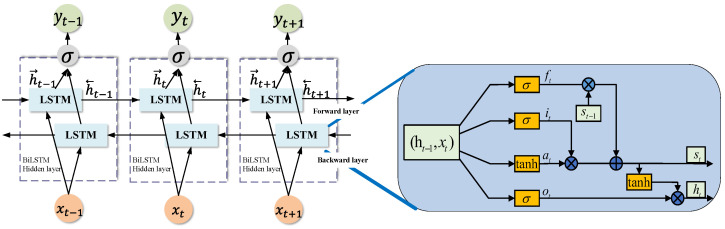
BiLSTM network structure.

As a fully connected neural network, BiLSTM uses the same back propagation, the same loss function, and the same optimizer. Different from other fully connected networks, BiLSTM introduces LSTM cells into the structural design, which can better capture the long-term dependencies in the sequence of NOMA. Figure 3 shows the overall structure of the BiLSTM and the LSTM cell unit structure in BiLSTM. The flow process of information flow in LSTM cells can be summarized from three door control equipments:

- Forget Gate
  (3) $$f_{t}=\sigma \left(W_{F}x_{t}+W_{f}h_{t-1}+b_{f}\right)$$
- Input Gate
  (4) $$\left\{\begin{array}{c} i_{t}=\sigma \left(W_{i}x_{t}+W_{i}h_{t-1}+b_{i}\right),\\ a_{t}=tanh\left(W_{a}x_{t}+W_{a}h_{t-1}+b_{a}\right) \end{array}\right.$$
- Output Gate
  (5) $$\left\{\begin{array}{c} o_{t}=\sigma \left(W_{o}x_{t}+W_{o}h_{t-1}+b_{o}\right),\\ h_{t}=o_{t}\cdot tanh\left(S_{t}\right) \end{array}\right.$$
- Cell status update
  (6) $$S_{t}=f_{t}S_{t-1}+i_{t}a_{t}$$

In the above equations, $h_{t-1}$ and $o_{t-1}$ are, respectively, the channel state and output of the previous cell. $h_{t}$ and $o_{t}$ are, respectively, the channel state and output of the current cell, and σ(·) is the Sigmoid activation function. Moreover, $W_{i},W_{i},W_{a},W_{o}$ are, respectively, the weights of each gate in the network, and $b_{f},b_{i},b_{a},b_{o}$ are, respectively, the bias terms of each gate. The process of finding partial derivatives with respect to cell state information can be expressed as

(7) $$\delta^{t-1}=\frac{\partial S^{t}}{\partial s^{t-1}}=\frac{\partial S^{t}}{\partial s^{t}}\frac{\partial s^{t}}{\partial s^{t-1}}=\delta^{t}\frac{\partial s^{t}}{\partial s^{t-1}}=\delta^{t}\left(f_{t}+\ldots \right).$$

When $f_{t}=1$, the remaining terms are small and will not affect the gradient conduction to the previous time instant. Therefore, the gradient will not disappear even if the network layer is deep. When $f_{t}=0$, the state at the current time will not be affected by that of the previous time, and the gradient will not be transmitted back, which can effectively solve the problem of gradient disappearance.

In addition to the network structure design, the BiLSTM network structure parameters for AMR of non-orthogonal signals will be taken into consideration, specifically including the following items:

(1) Bidirectional LSTM Layers: The number of Bidirectional LSTM Layers refers to the number of convolutional layers where the input symbol sequences pass through. In this work, the modulation recognition performance is observed through subsequent simulation experiments under different LSTM layer number designs, and the maximum number of layers is set to three layers according to experience.

(2) Forgetting Bias: Forgetting Bias is designed to reduce the scale of forgetting during training. The activation function Sigmoid of the forgetting gate has the characteristic that the larger the input is, the closer the output is to 1. Therefore, if there is a fixed internal bias, the overall input will not be too small. Under such conditions, the network can not only remember more previous information from NOMA signals but also reduce the occurrence of the vanishing gradient problem to some extent. In the subsequent simulation, the modulation recognition performance with a Forgetting Bias of 1, 0.8, and 0.6 will be tested, respectively.

(3) Time Step: Time Step is determined by the dimension of the input data. Then the IQ data of a NOMA symbol will be input at each Time Step. The modulation recognition performance in the presence of 4, 8, and 12 symbols in the input NOMA signal block will be simulated, respectively, so as to determine the optimal number of group packets. In the subsequent simulation, the modulation recognition performance when there are 4, 8, and 12 NOMA symbols in the input packet will be tested, respectively, so as to determine the optimal value of the Time Step.

The above network structure design will be simulated in Section 5.1, referring to the current commonly used settings of neural networks, and proposing the optimal network architecture. The specific network structure selection is shown in the Table 1.

### 3.2. Deep Transfer Learning Enhanced BiLSTM

In many practical communication scenarios, it is difficult to obtain enough labeled data for training, which will cause difficulties in feature extraction, such as overfitting [29]. However, transfer learning can effectively reduce the requirements of data volume in a NOMA system and training time in the target domain [30]. This subsection will carry out modulation recognition research on non-orthogonal signals in small sample scenarios to expand the application scope of the modulation recognition method based on BiLSTM. We extend the study to the case where only a small number of labeled signal samples can be obtained in the target scenario, while a large number of labeled signal samples can be obtained in other scenarios.

Domain and task are two important concepts in the transfer learning model. The domain consists of a source domain and a target domain. Given the domain *D*, the task *T* consists of a category space *Y* and a prediction function f(x), that is T={Y,f(x)}. Considering only one source domain $D_{S}$ and one target domain $D_{T}$ in this work, the source domain data and the learning task can be expressed as $D_{S}=\left\{\left(x_{S_{1}},y_{S_{1}}\right),\ldots ,\left(x_{S_{n_{S}}},y_{S_{n_{S}}}\right)\right\}$ and $T_{s}$, respectively. $x_{S_{i}}\in X_{S}$ denotes the data instance, and $y_{S_{i}}\in Y_{S}$ denotes the modulation category corresponding to the data. Referring to the representation of the source domain, the target domain data and the learning task can be expressed as $D_{T}=\left\{\left(x_{T_{1}},y_{T_{1}}\right),\ldots ,\left(x_{T_{n_{T}}},y_{T_{n_{T}}}\right)\right\}$ and $T_{T}$, respectively.

Transfer learning relaxes the assumption that the training data must be independent and identically distributed with the test data and does not need to train the model in the target domain from scratch. Therefore, the layer transfer model will be used in the pre-trained BiLSTM model so as to transfer a part of the network structure and parameters in the source domain to a part of the target domain neural network. Then the knowledge of $D_{T}$ and $T_{S}$ can be exploited to achieve the goal of improving the statistical performance under the learning task $T_{T}$, which satisfies $D_{S}\neq D_{T}$ or $T_{S}\neq T_{T}$.

**Figure 4:**
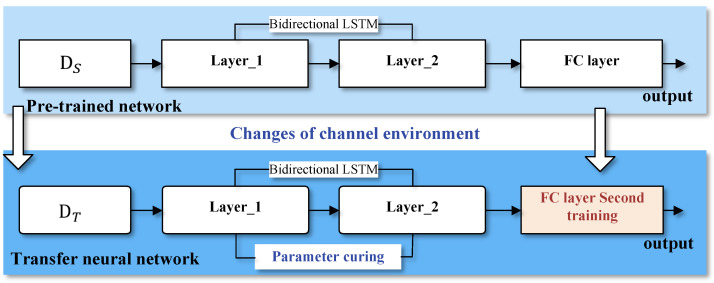
Structure of transfer learning model with layer transfer.

The model based on layer transfer is shown in Figure 4. The structure and parameters trained in the source domain are copied, and the layer without copy is trained with the NOMA sequence information of the target domain. The implementation process of the model can be divided into the following steps:

(1) Step 1: Set the NOMA sequence information of the source domain and target domain as *S* and *T*, respectively. Use the data of *S* to train the BiLSTM network and obtain the training model $H_{S}$.

(2) Step 2: Use the part layers parameters of the model $H_{S}$ in $H_{T}$ and solidify the parameters.

(3) Step 3: Use the data of *T* to retrain the parameters of unsolidified network layers in model $H_{T}$.

When the curing layer and the secondary training layer are selected, different choices affect the recognition accuracy of small sample non-orthogonal signals in the downlink system. The mode of curing can be generally divided into three cases: ① *Directly train the original model twice without curing layer*. ② *Solidify all bidirectional LSTM layers and train the fully connected layer twice*. ③ *Solidify the shallow bidirectional LSTM layer and the deep bidirectional LSTM layer, then train the fully connected layer twice*. In order to determine the best model architecture, this work will simulate the performance of the above three different layer migration models in Section 5.1.

## 4. Proposed Attention-Based Spatio-Temporal Fusion Network for Uplink AMR

In the uplink non-orthogonal transmission scenario, the number of modulation schemes increases exponentially with the number of users. Constellation mapping recognition methods struggle to classify the modulation by extracting features. We consider utilizing the strong ability of DL to fit features of NOMA signals, and extracting features from the spatial and temporal dimensions. In Section 4.1, we present the design of STFAN. The corresponding DL optimization method is illustrated in Section 4.2.

### 4.1. Proposed Neural Network Architecture

**Figure 5:**
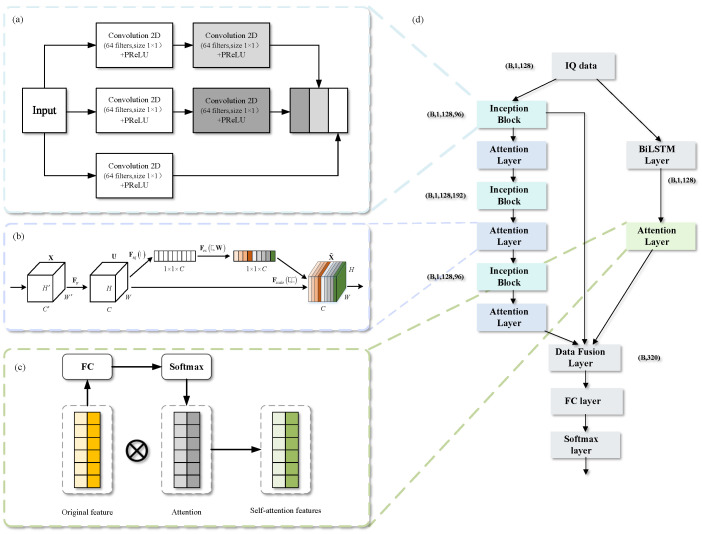
Spatio-temporal fusion network architecture based on attention mechanism. (**a**) Structure of Inception. (**b**) Structure of SENet. (**c**) Self-attention feature implementation of BiLSTM. (**d**) Structure of STFAN.

In this subsection, the components of STFAN are introduced, including spatial feature extraction, temporal feature extraction, and feature fusion. The specific structure of the network is shown in Figure 5.

#### 4.1.1. Spatial Feature Extraction

The spatial feature extraction of STFAN is composed of three layers of Inception blocks and Attention layers. The Inception block extracts signal space information, and the Attention layer implements recalibration for different features of signals. The shortcut structure is used to fuse the shallow information with the deep information to prevent information loss. The specific structure is shown in Figure 5d, on the left side of the framework.

Google has taken the lead in proposing Inception [31], which can not only maintain the sparsity of the network structure, but also take advantage of the high computational performance of the dense matrix. The core contribution of Inception in this work is to reduce the computational complexity and perform convolutional reaggregation at multiple signal dimensions simultaneously. Convolution at multiple scales simultaneously can effectively extract the multi-dimensional features of the signal, which leads to higher accuracy in classification judgments. Figure 5a shows the detailed structure of Inception adopted in this paper, which contains a total of three layers. The first layer uses a larger 1×8 convolution kernel, and the second layer uses a smaller 1×3 convolution kernel. The third layer outputs directly, then the fusion module of Inception parallelizes the output of the three layers in the depth direction of the network.

The output of each Inception layer is connected to the Attention layer separately. In this work, SENet [32] is used as the Attention layer, which has the advantages of easy implementation, good effect, and low complexity. The complexity of NOMA signals is higher than that of traditional OFDM signals, which results in difficulty in selecting out the key feature channels. SENet can learn the correlation between channels, screen out the channel-specific attention, and assign different weights. The structure of SENet is shown in Figure 5b. The implementation process of SENet can be divided into three operations:

(1) Squeeze Operation: The global average pooling is used to obtain the global receptive field in the channel dimension.

(2) Excitation Operation: A fully connected layer and a Softmax layer are used to model the correlation between channels. The weight of the Excitation output represents the importance of each feature channel.

(3) Reweight Operation: By using multiplication, each channel is weighted to the original feature to realize the re-calibration of the original features in NOMA signals.

#### 4.1.2. Temporal Feature Extraction

The temporal feature extraction module of STFAN consists of the BiLSTM layer and Attention layer, as shown on the right of Figure 5d. The received I/Q data are expanded into a sequence of symbols based on the time dimension. By mining the temporal relationship of symbols in the sequence, it is beneficial to extract new signal features and further strengthen the recognition ability of multiclass classification in the uplink system.

BiLSTM is used in the temporal feature extraction module to extract features in the time dimension of I/Q signals waiting for recognition, which is the same as the one described in [33]. Each feature point on the feature map output by the BiLSTM layer has different contribution values to the modulation recognition performance. Therefore, the self-attention mechanism is introduced at the end of BiLSTM as the Attention layer.

The self-attention mechanism is a variant of the attention mechanism, which can effectively reduce the dependence on external sequence information and is better at mining the internal correlation of signal features. As the self-attention feature implementation process of BiLSTM shown on the left of Figure 5c, the similarity of the query and each key will be calculated using the fully connected layer to obtain the weight. Then the self-attention feature map of BiLSTM will be obtained by multiplying the weights normalized by the Softmax function to the original feature map.

#### 4.1.3. Feature Fusion

After the network extracts the features in the temporal and spatial domains, the above features are fused together using the data fusion layer. In the data fusion layer, the shallow and deep spatial features are globally pooled to obtain a one-dimensional vector. Then the final feature is obtained by fusing the obtained temporal features, and then it is passed through the fully connected layer and the Softmax layer to output the final recognition result. The fusion of spatial and temporal features can amplify the difference between signals, which can improve recognition accuracy.

### 4.2. DNN Parameter Optimization

The performance of deep learning is closely related to optimization algorithms and data processing. Different methods will lead to different training effects [34]. Several parameter optimizations used in STFAN will be described as follow:

(1) Data Normalization: Data normalization can accelerate the convergence speed of the network and improve the accuracy of the model to a certain extent. Common data normalization methods include max-min normalization and Z-score normalization, etc. Z-score normalization will be used in STFAN to set the mean of the signal to 0 and the standard deviation to 1, which is calculated as

(8) $$x^{*}=\frac{x-\bar{x}}{\sigma },$$

where $\bar{x}$ denotes the mean, σ denotes the standard deviation, and *x* is the vector of each eigenvalue.

(2) Batch Normalization: Covariate Shift (ICS) can lead to a lower learning rate and convergence speed of the network. Batch Normalization (BN) is an optimization method proposed by Google in 2015 to solve ICS [35]. BN is the normalization and decentralization of Batch data, which can reduce the dependence on parameter initialization, accelerate the training process, and increase the generalization ability of the model. The specific implementation process can be expressed as

(9) $$y_{i}=\gamma {\hat{x}}_{i}+\beta ,$$

where

(10) $${\hat{x}}_{i}=\frac{x_{i}-\mu_{B}}{\sqrt{{\sigma_{B}}^{2}+\varepsilon }}.$$

In Equation (10), $x_{i}$ denotes the *i*-th data sequence in a single Batch, $\mu_{B}$ denotes the mean of the data in a single Batch, and ${\sigma_{B}}^{2}$ denotes the variance of a single Batch. The parameters γ and β in Eqiation (9) are introduced to restore the expressive power of the data itself. When $\gamma^{2}=\sigma^{2}$ and β=μ, the equivalent transformation can be achieved with the preservation of the distribution in original features. Moreover, the convergence speed of STFAN will be accelerated after the introduction of the BN layer.

(3) Activation Function: Since the input I/Q symbol data signal have negative values, the PReLU activation function will be introduced in this subsection as an alternative [36]. The slope of PReLU is small in the negative value domain, which can effectively avoid the ‘Dead ReLU’ phenomenon. Compared with ELU, PReLU is a linear operation in the negative range with a small but never zero outcome. The specific expression of PRELU can be expressed as

(11) $$f\left(y_{i}\right)=\left\{\begin{array}{cc} y_{i},\;\; & \mathrm{if}y_{i}\geq 0,\\ a_{i}y_{i},\;\; & \mathrm{if}y_{i}\leq 0. \end{array}\right.$$

where $a_{i}$ is generally between 0 and 1. If $a_{i}=0$, then *f* will become a ReLU. If $a_{i}$ is a fixed decimal close to 0, then *f* will become a Leaky ReLU. The optimal value, $a_{i}=0.27$, is determined by experience and multiple experiments.

## 5. Simulation Results and Analysis

In this section, we implement the proposed BiLSTM-based deep transfer learning network in the downlink non-orthogonal system and STFAN in the uplink non-orthogonal system.

### 5.1. Downlink Non-Orthogonal System

In downlink non-orthogonal system, the modulation recognition performance of BiLSTM in Section 3.1 is simulated. The structural parameters of the model are determined, respectively, and the performance of modulation recognition in the Gaussian channel and non-ideal channel with random bias is studied. Furthermore, the deep transfer model proposed in Section 3.2 is simulated, and the performance is analyzed in three different layer transfer models under a Gaussian channel.

#### 5.1.1. Dataset and Parameters

This dataset contains a total of five different non-orthogonal modulation methods, namely SCMA, MUSA, PDMA, WSMA, and PD-NOMA. Each scheme generates 960,000 NOMA symbols by Monte Carlo simulation, and the noise added by each NOMA symbol obeys Gaussian distribution under a Gaussian channel. In order to make full use of the characteristics of the Gaussian noise partially superimposed region, 12 NOMA symbols are formed into a NOMA signal block. Therefore, there are a total of 80,000 NOMA signal blocks for each method in the training set, where 80% of the samples are used as the training set, and 20% of the samples are used as the cross-validation set. Each NOMA signal block length *L* satisfies L=96.

Under Gaussian channel, the *n*-*th* baseband symbol sequence model can be expressed as

(12) $$y\left(n\right)=\sum_{j=1}^{J}s_{j}x_{j}\left(n\right)+v\left(n\right),$$

where *n* satisfies n∈[1,L], v(n) denotes Gaussian white noise, which satisfies mean zero and variance $\theta^{2}$. The SNR of symbol sequences of each modulation in the training set satisfies the uniform distribution of [−10 dB, 20 dB]. Concurrently, the Monte Carlo Simulation is also used to generate 2000 symbol sequences corresponding to the SNR of each modulation signal in the test set. The SNR range of the test set is [−10 dB, 20 dB] with an interval of 2 dB.

Under the channel with random bias, the first baseband symbol sequence model can be expressed as

(13) $$y\left(n\right)=e^{j(\theta +2\pi fn)}\sum_{j=1}^{J}s_{j}x_{j}\left(n\right)+v\left(n\right),$$

which satisfies n∈[1,L], θ∈[0,20] and *f* = 1 × 10^−3^. The subsequent training set and test set settings are consistent with the Gaussian channel case. Additionally, the remaining parameters of the downlink non-orthogonal signal system are listed in Table 2.

For the simulation based on transfer learning, the number of training samples corresponding to each modulation is $N_{S}=$ 80,000 in the source domain, compared to $N_{T}=400$ in the target domain. In the pre-training stage, the dataset with small bias is used for the source domain signals under Gaussian channel, where the maximum phase bias and the frequency bias are $20^{\circ }$ and 1 $\times \;10^{-4}$, respectively. In the transfer learning stage, the dataset with large bias is used for the target domain signals, where the maximum phase bias and frequency bias are $40^{\circ }$ and 1 $\times \;10^{-3}$, respectively. In the testing phase, the signal type of the dataset used is the same as the target domain, and the SNR range of the test set is [−10 dB, 20 dB]. For each signal category, 1000 NOMA symbols are generated by Monte Carlo simulation.

**Figure 6:**
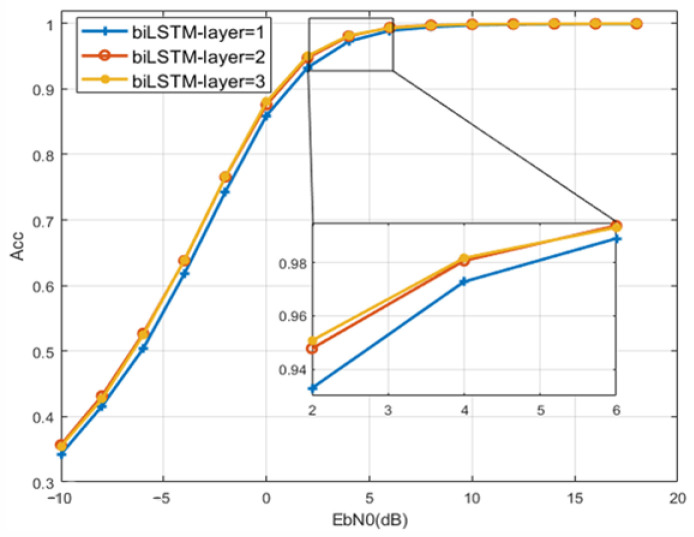
Network performance comparison with different LSTM layer.

**Figure 7:**
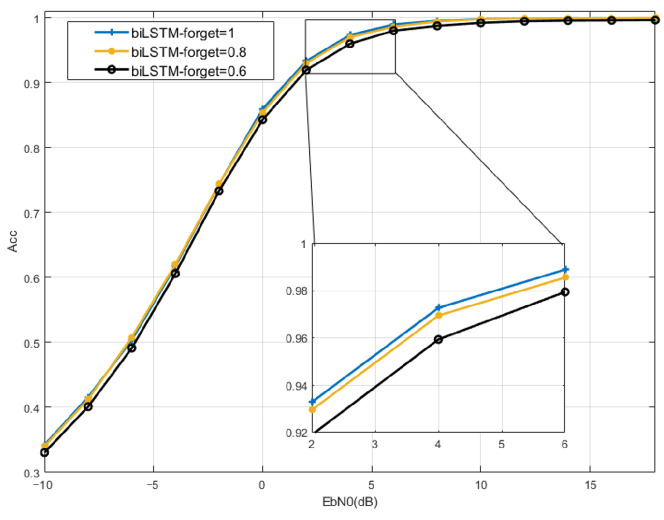
Network performance comparison with different forgetting bias.

**Figure 8:**
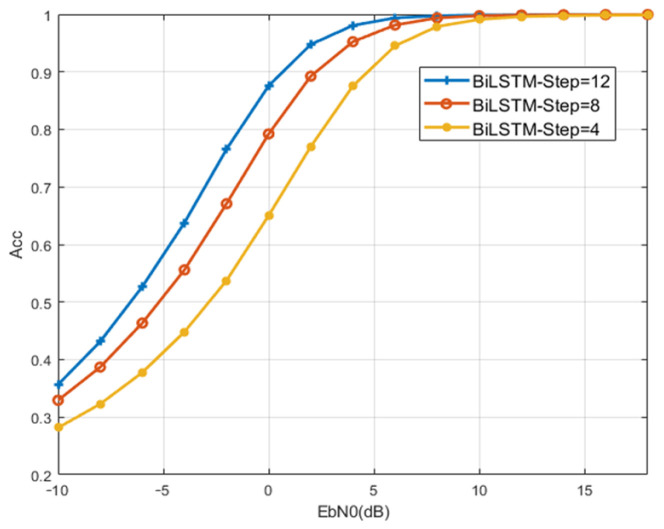
Network performance comparison with different Time Step.

To select appropriate structural parameters of BiLSTM, the recognition accuracy will be compared with different values. Simulation results about the number of layers of bidirectional LSTM are shown in Figure 6. When the number of layers is two or three, the recognition accuracy of BiLSTM is higher. Considering the network complexity and recognition performance, the number of bidirectional LSTM layers of the BiLSTM model is set to two layers. Figure 7 shows the simulation results related to the Forgetting Bias. Considering that a large bias can effectively prevent the occurrence of gradient disappearance, the Forgetting Bias is designed to be 1 for its recognition performance. Figure 8 shows the simulation results for different numbers of Time Step cases. When the SNR is 0 dB, the recognition accuracy is 10% higher than that of 8 and 20% higher than that of 4 when the number of Time Steps is 12. Considering that increasing the number of Time Steps will increase the complexity of the network and increase the burden of hardware computing, the Time Step of this model is set to 12. As the computation complexity shown in Table 3, the overall parameters except for FLOPS of BiLSTM are lower than those of baseline models for the simple structure of FC-DNN. Regarding learning speed, the BiLSTM model is considered the fastest as it takes 489 s per epoch. The prediction time of BiLSTM is 173 μ which is lower than that of FC-DNN and LSTM, which proves BiLSTM can achieve low complexity with fewer parameters and shorter training time.

Moreover, multi-class cross-entropy is the loss function of BiLSTM. The optimizer, learning rate, and decay value of the learning rate are set to Adam, 0.0001, and 0, respectively. The Batch size in the training process is 128, and the data inside the Batch will be shuffled and re-selected after each epoch. Additionally, the training stop condition is when the training number reaches 40 epochs or when the loss value of the validation set does not decrease for 10 consecutive epochs.

#### 5.1.2. Gaussian Channel

**Figure 9:**
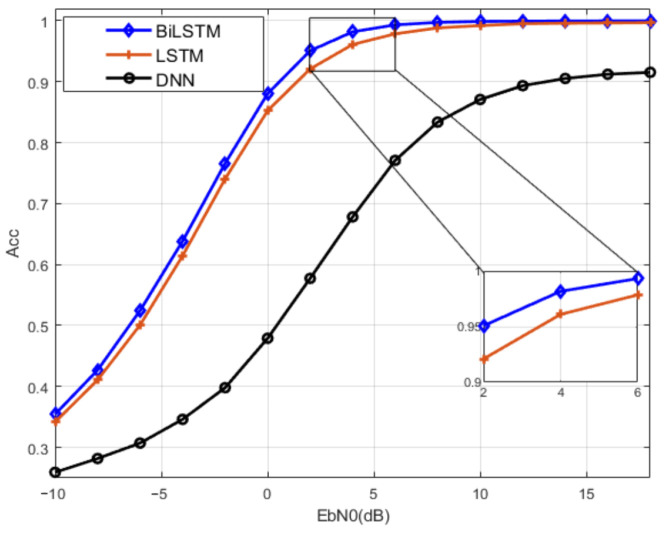
Comparison of recognition accuracy of BiLSTM under Gaussian channel.

In this part, the performance of modulation recognition of the BiLSTM network under Gaussian channel is studied. The unidirectional LSTM network and FC-DNN are used as performance comparison and the obtained results are shown in Figure 9.

RNN has a stronger ability to extract features for long sequences than FC-DNN. The modulation recognition accuracy obtained by BiLSTM and LSTM have better performance than that of the FC-DNN with SNR in the range of [−10 dB, 10 dB]. Compared with LSTM, BiLSTM can accurately describe the essential characteristics of the signal by fitting features in both forward and backward directions, which can obtain better performance for all SNR. The recognition accuracy of LSTM is 85% at 0 dB, but the recognition accuracy of BiLSTM network is close to 90% at 0 dB, even close to 100% at 6 dB.

**Figure 10:**
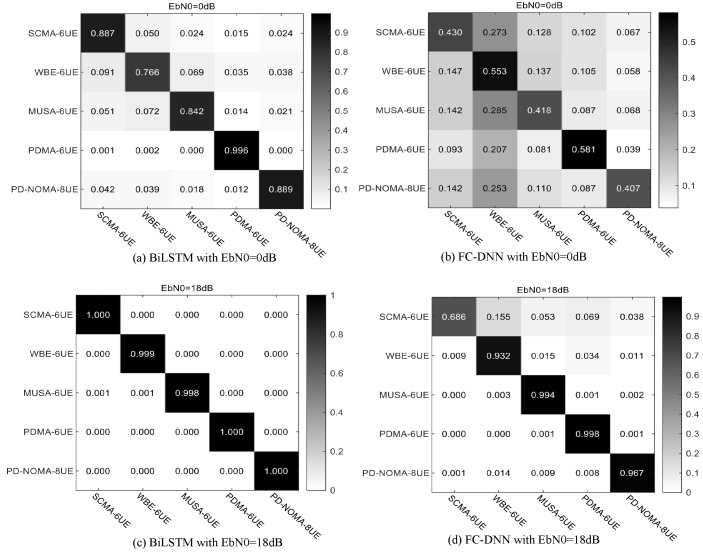
Confusion matrix for MR of BiLSTM and FC-DNN under Gaussian channel.

As the confusion matrix shown in Figure 10, the modulation recognition performance of BiLSTM is far better than that of FC-DNN. When EbN0 is 0 dB, the BiLSTM can identify a non-orthogonal modulation method with an accuracy of at least 75%, even at 0099.6% for PDMA. However, the accuracy of FC-DNN for various methods is only about 50%, and the network can be greatly disturbed by noise. When EbN0 is 18 dB, the recognition accuracy of BiLSTM for each method is above 99.8%. Additionally, the recognition rate of FC-DNN is only 92%, which is due to the poor recognition of SCMA.

**Figure 11:**
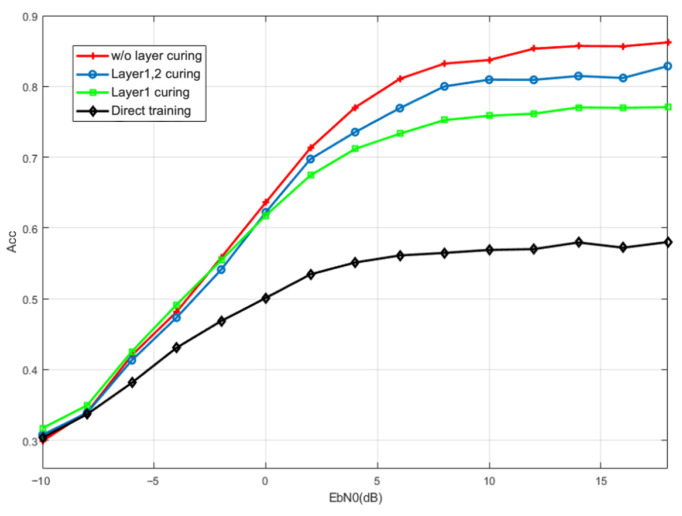
Comparison of recognition performance of solid transfer models at different layers.

The performance of the BiLSTM with transfer learning under a Gaussian channel is shown in Figure 11. Directly retraining the model requires a large amount of training data, which leads to the incomplete convergence of the network and the poor generalization of the network. Therefore, the worst transfer learning model has 20% better recognition accuracy at 20 dB than the directly trained model. Concurrently, the features extracted in the old training environment are also applicable to the new training environment to some extent. The overall fine-tuning performance of the trained model is better than the other two transfer learning models, and its recognition accuracy can reach 86% at 20 dB. Among different solidified layers, the second bidirectional LSTM layer extracts deep features based on the shallow features extracted from the first layer. The mismatch between the features extracted by the old channel shallow network and the new channel data results in the poor recognition performance of curing the first layer.

#### 5.1.3. Channel with Random Phase Bias

**Figure 12:**
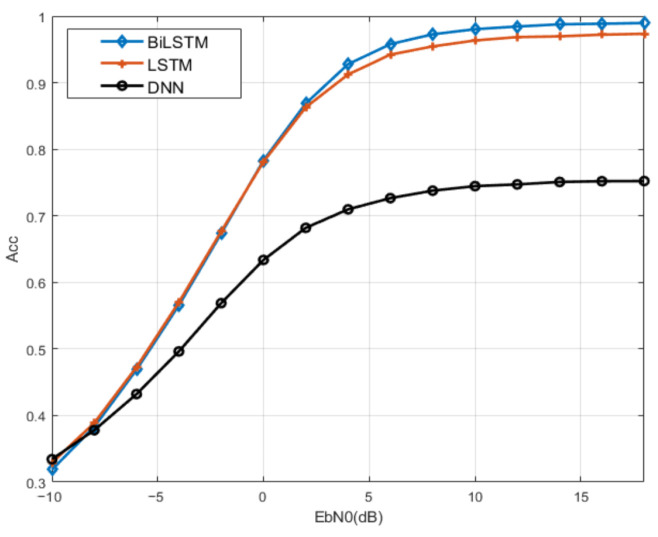
Comparison of recognition accuracy of BiLSTM under channel with random bias.

The modulation recognition performance of BiLSTM is studied under the channel with phase bias and frequency bias. From the comparison results shown in Figure 12, the recognition rate of BiLSTM and LSTM can still reach more than 95% at high SNR. Concurrently, BiLSTM has a stronger ability to extract network features, and the performance of BiLSTM is about 3% higher than that of LSTM. However, the recognition rate of FC-DNN is only 75%, which indicates that BiLSTM has better robustness.

**Figure 13:**
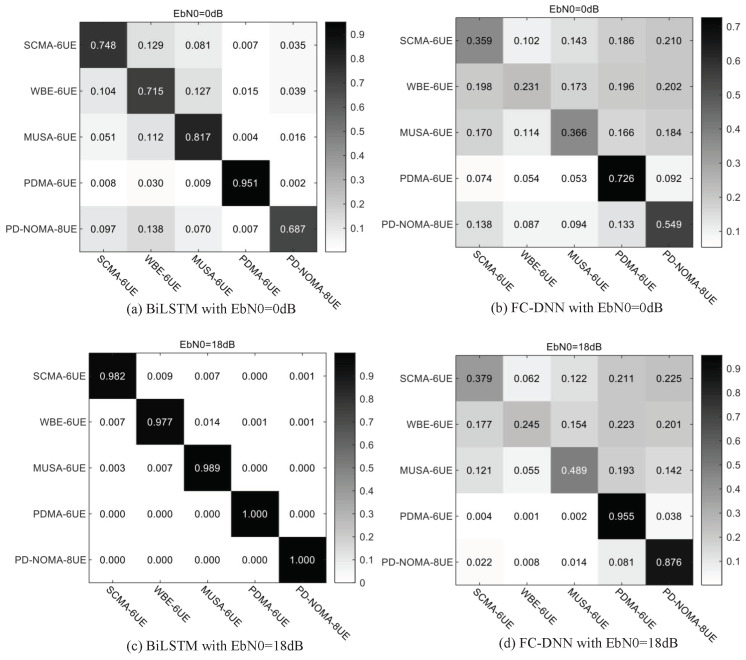
Confusion matrices for MR of BiLSTM and FC-DNN under channel with random bias.

As the confusion matrix results shown in Figure 13, BiLSTM for baseband symbol sequences has strong recognition ability and robustness. When EbN0 is 0 dB, BiLSTM can identify the above five non-orthogonal modulation methods with high accuracy. When EbN0 is 18 dB, all the methods can be recognized with nearly 100% recognition accuracy. As a comparison, the recognition performance of FC-DNN is extremely poor at low SNR, and has no significant improvement for SCMA, WSMA, and MUSA at high SNR.

### 5.2. Uplink Non-Orthogonal System

In an uplink non-orthogonal system, the modulation recognition performance of STFAN is simulated for 2ASK, QPSK, and 16QAM superimposed signals, and the performance is compared with that of the traditional deep networks.

#### 5.2.1. Dataset and Parameters

The dataset used in this simulation is the baseband symbol sequence obtained after pre-processing the IF signal received by the base station, and the base station has only one receiving antenna. A total of three users with different distances randomly choose from {2ASK, QPSK, and 16QAM} and send signals to the base station at the same time, and the signals of each user are completely synchronized. Since the three users can choose the modulation mode randomly, there will be 27 modulation modes under different powers combined with the transmit power. Each modulation mode contains a total of 40,000 symbol sequences, which contain 2×128 numbers. The total number of symbol sequences in this dataset is 1,080,000, of which 85% of the symbol sequences will be used as the training set and 15% of the symbol sequences will be used as the validation set.

Under Gaussian channel, the *i*-th baseband symbol sequence can be given by

(14) $$r\left(i,n\right)=P_{A}x_{A}\left(i,n\right)+P_{B}x_{B}\left(i,n\right)+P_{C}x_{C}\left(i,n\right)+v\left(i,n\right),$$

where *n* satisfies n=1,K,100,v(i,n):CN(0,1). The Gaussian noise added to the training set and the test set is exactly the same as the configuration of downlink non-orthogonal system. Concurrently, each symbol sequence has the same power and different noise internally.

Under the channel with random bias, the phase bias in the transmission sequence of the three users is randomly distributed within the range of [0,$\theta_{max}$]. The *i*-th baseband symbol sequence can be given by

(15) $$r\left(i,n\right)=e^{j\theta_{A}}P_{A}x_{A}\left(i,n\right)+e^{j\theta_{B}}P_{B}x_{B}\left(i,n\right)+e^{j\theta_{C}}P_{C}x_{C}\left(i,n\right)+v\left(i,n\right),$$

where *n* satisfies n=1,K,100,v(i,n):CN(0,1), and $\theta_{A},\theta_{B},\theta_{C}$ represent the phase bias of each user, respectively. The Gaussian noise added to the training set and the test set is exactly the same as the configuration of the Gaussian channel. The remaining parameters of the uplink non-orthogonal signal model are listed in Table 4.

As the computation complexity shown in Table 5, the FLOPS of STFAN is higher than that of ResNet and CNN. With more parameters and layers than CNN and ResNet, STFAN requires a training period longer than other models. STFAN has a more complex network structure. In order to extract the temporal and spatial features of superimposed signals, STFAN sacrifices computational complexity to achieve far better accuracy.

#### 5.2.2. Gaussian Channel

**Figure 14:**
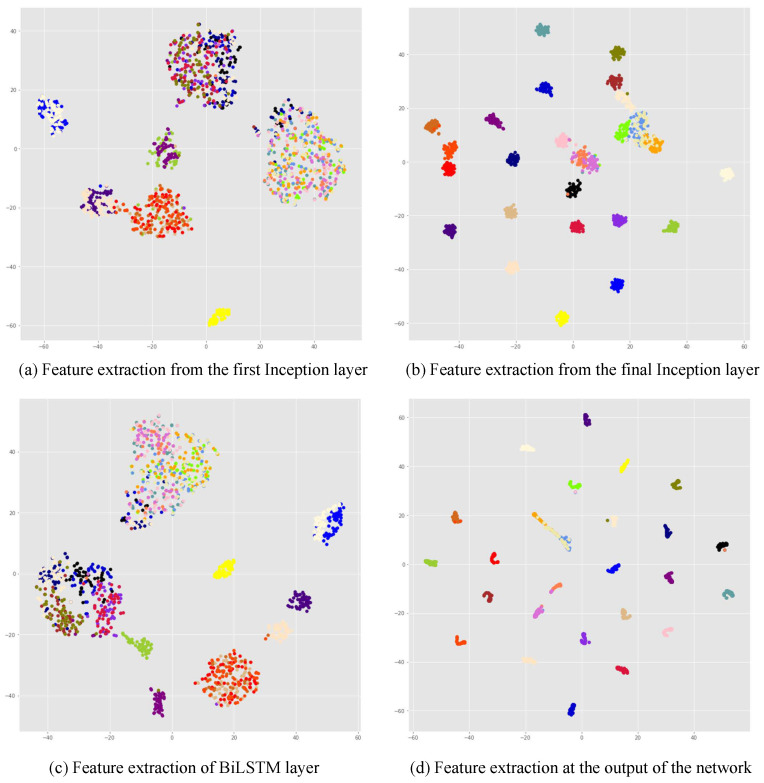
Visualization of features extraction.

This part focuses on the modulation recognition performance of STFAN in Gaussian channels. When the SNR is 20 dB, the visualization of features extracted by STFAN is shown in Figure 14. Different colored dots in the figure represent signals with different modulation modes. Figure 14a,b shows that a deeper Inception layer can lead to more obvious differences between different signals. Figure 14c shows that the BiLSTM layer is not as strong in distinguishing signals as the Inception layer, but the BiLSTM layer can supplement the signal features that cannot be extracted by the Inception layer. In Figure 14d, the signal features basically have no overlap after feature fusion.

**Figure 15:**
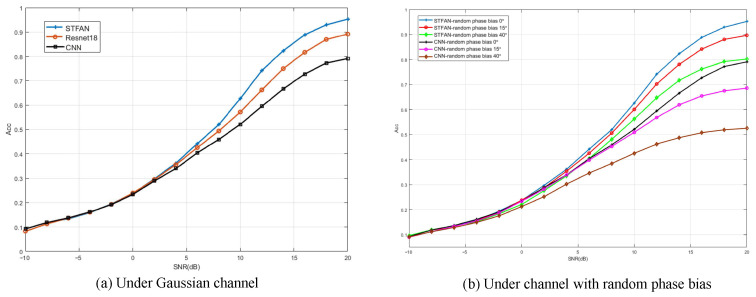
Performance of STFAN.

Compared with CNN and ResNet18, the results are shown in Figure 15a. The recognition accuracy of STFAN, 18-layer ResNet, and CNN are basically the same under low SNR. However, the gap between the recognition accuracy of STFAN and the other two schemes gradually increases with the increase of SNR. When the SNR is 20 dB, the recognition rate of STFAN can reach 96.6%, compared to 89.35% in ResNet18, and 79.25% in CNN. The main reasons for the above performance difference are the Inception structure and the BiLSTM layer used in STFAN, which can extract the spatial and temporal features of the network completely and efficiently. Moreover, the attention mechanism of STFAN can enhance the ability to extract effective features and further improve recognition accuracy.

**Figure 16:**
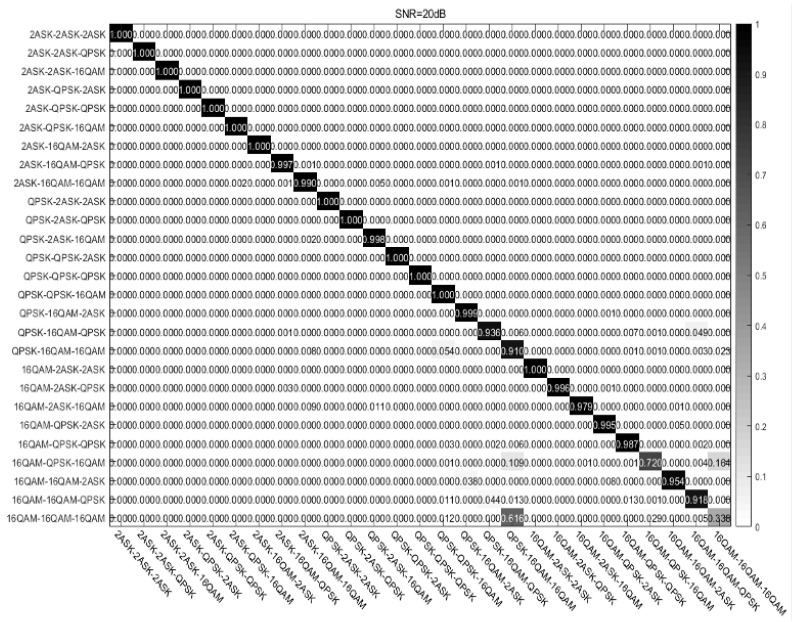
Classification confusion matrix at SNR of 20 dB.

In order to further study the recognition performance difference of STFAN on different modulation signal modes, the confusion matrix shown in Figure 16 is plotted for an SNR of 20 dB. Among the 27 classes of modulation modes, the recognition accuracy of 25 classes is above 90%, and the recognition accuracy of 22 classes is above 95%. Whereupon, the recognition effect can meet the basic requirements of data transmission in the Internet-of-Things scenario. Moreover, the reason that limits the overall recognition performance is mainly due to the poor recognition of the 16QAM-16QAM-16QAM mode. For the lower power of 16QAM, the distance between 16QAM and QASK constellation points is very small, which can result in misjudgment of STFAN.

#### 5.2.3. Channel with Random Phase Bias

As the result shown in Figure 15b, the performance of STFAN is still better when the three users have random bias. Especially in the small phase bias scenario, the recognition accuracy is almost the same as the performance without random phase bias in the interval range of SNR less than 2 dB. At a SNR of 20 dB, the recognition performance is only 5% different from the former, and the accuracy can still reach 90%. When the random phase bias distribution is in [0, 40], the recognition accuracy based on STFAN can reach 80%, compared to that of 53% in CNN. The above results reflect that the network has a strong feature extraction ability, which can extract the phase change characteristics of the received data. Additionally, STFAN has strong robustness, which can adapt to complex channel scenarios.

## 6. Conclusions

In this work, AMR is first introduced into non-orthogonal signal recognition. In the downlink scenario, a non-orthogonal recognition model based on BiLSTM is proposed to mine the sequence information in the superimposed signal. Additionally, in the case of insufficient samples in real scenes, deep transfer learning is introduced. In the uplink scenario, we innovatively propose a spatio-temporal feature extraction method. The Inception module is used to extract the spatial features of the signal, the BiLSTM layer is used to extract the temporal features of the signal, and the attention mechanism is embedded to further improve the effectiveness of the features. Simulation results show that the algorithms in different scenarios maintain high accuracy and high robustness. Specifically, compared to existing modulation recognition methods in the downlink scenario, BiLSTM can achieve more than 25% accuracy gain over FC-DNN under high SNR conditions of Gaussian channels. In the uplink scenario, STFAN is superior to ResNet and CNN under different channel conditions, especially the accuracy of the algorithm can reach 96.6% at 20 db of Gaussian channel. For future work, further improvements can be made in the separation and identification of superimposed signals.

## Figures and Tables

**Table 1:** Network Structure Selection of BiLSTM.

Name	Parameters	Activation Function	Output Data Stream	$N_{p}$ ^1^
Input Layer	/	/	(128,2)	/
BiLSTM_1	$N_{FL}=128,N_{BL}=128$ ^2^	Tanh/Sigmoid	(12,256)	140,288
BiLSTM_2	$N_{FL}=64,N_{BL}=64$	Tanh/Sigmoid	(1,128)	164,352
FC layer	64	ReLU	(1,64)	8256
Output Layer	5	Softmax	5	325

${}^{1}$ $N_{p}$ denotes the number of parameters. ^2^ $N_{FL}$ is the number of forward LSTM neurons, and $N_{BL}$ is the number of backward LSTM neurons.

**Table 2:** Parameters for Downlink Non-orthogonal Signal System.

Parameters Name	Parameters Value
Multiplexing Type	SCMA, MUSA, PDMA, PD-NOMA, WSMA
Carrier Frequency	1.2 GHz
Symbol Rate	15.36 MHz
Sampling Rate	122.88 MHz
$N_{I/Q}$ ${}^{1}$	128
$N_{U}$ ${}^{2}$	6
$N_{R}$ ${}^{2}$	4
Oversampling Rate	1.5
SNR Range	−10 dB∼20 dB

^1^ $N_{I/Q}$ denotes the complex baseband I/Q data points per path. ^2^ $N_{U}$ is number of users, and $N_{R}$ is number of resources.

**Table 3:** Computation Complexity Comparison between STFAN and baseline models for downlink systems.

Models	FC-DNN	LSTM	BiLSTM
Total parameters	822,533	797,957	313,221
Epochs	50	30	30
Training time (s)/epoch	602	524	489
Prediction time (μs)/sample	192	175	173
FLOPS	19,784,965	191,868,832	59,445,664

**Table 4:** Parameters for Uplink Non-orthogonal Signal System.

Parameters Name	Parameters Value
Modulation Type	2ASK, QPSK, 16QAM
Carrier Frequency	1.2 GHz
Symbol Rate	15.36 MHz
Sampling Rate	122.88 MHz
$N_{I/Q}$	128
$P_{A}/P_{B}/P_{C}$	0.2/0.3/0.5
$N_{U}$	3
$N_{R}$	1
Oversampling Rate	3
SNR Range	−10 dB∼20 dB

**Table 5:** Computation Complexity Comparison between STFAN and baseline models for uplink systems.

Models	CNN	ResNet	STFAN
Total parameters	8,593,563	4,214,922	23,370,448
Epochs	50	40	40
Training time (s)/epoch	631	598	721
Prediction time (μs)/sample	193	188	189
FLOPS	81,259,328	158,353,019	273,799,168

## Data Availability

Not applicable.
